# Viral infections in critical care: a narrative review

**DOI:** 10.1111/anae.15946

**Published:** 2023-01-12

**Authors:** A. Conway Morris, A. Smielewska

**Affiliations:** ^1^ Division of Anaesthesia, Department of Medicine University of Cambridge UK; ^2^ John V Farman Intensive Care Unit Addenbrooke's Hospital Cambridge UK; ^3^ Department of Clinical Virology, LCL, CSSB Liverpool University Hospitals NHS Foundation Trust Liverpool UK; ^4^ School of Clinical Medicine University of Liverpool UK

**Keywords:** blood borne viruses, emerging viruses, pneumonia, respiratory viruses

## Abstract

Viral infections form a substantial part of the intensive care workload, even before the recent and ongoing COVID‐19 pandemic. The growing availability of molecular diagnostics for viral infections has led to increased recognition of these pathogens. This additional information, however, provides new challenges for interpretation and management. As the SARS‐CoV‐2 pandemic has amply demonstrated, the emergence and global spread of novel viruses are likely to provide continued challenges for critical care physicians into the future. This article will provide an overview of viral infections relevant to the critical care physician, discussing the diagnosis and management of respiratory viral infections, blood borne and enteric viruses. We will also discuss herpesviridae complications, commonly seen due to reactivation of latent infections. Further, we explore some rarer and emerging viruses, including recognition of viral haemorrhagic fevers, and briefly discuss post‐viral syndromes which may present to the intensive care unit. Finally, we will discuss infection control and its importance in preventing nosocomial viral transmission.

## Introduction

**Figure 1:**
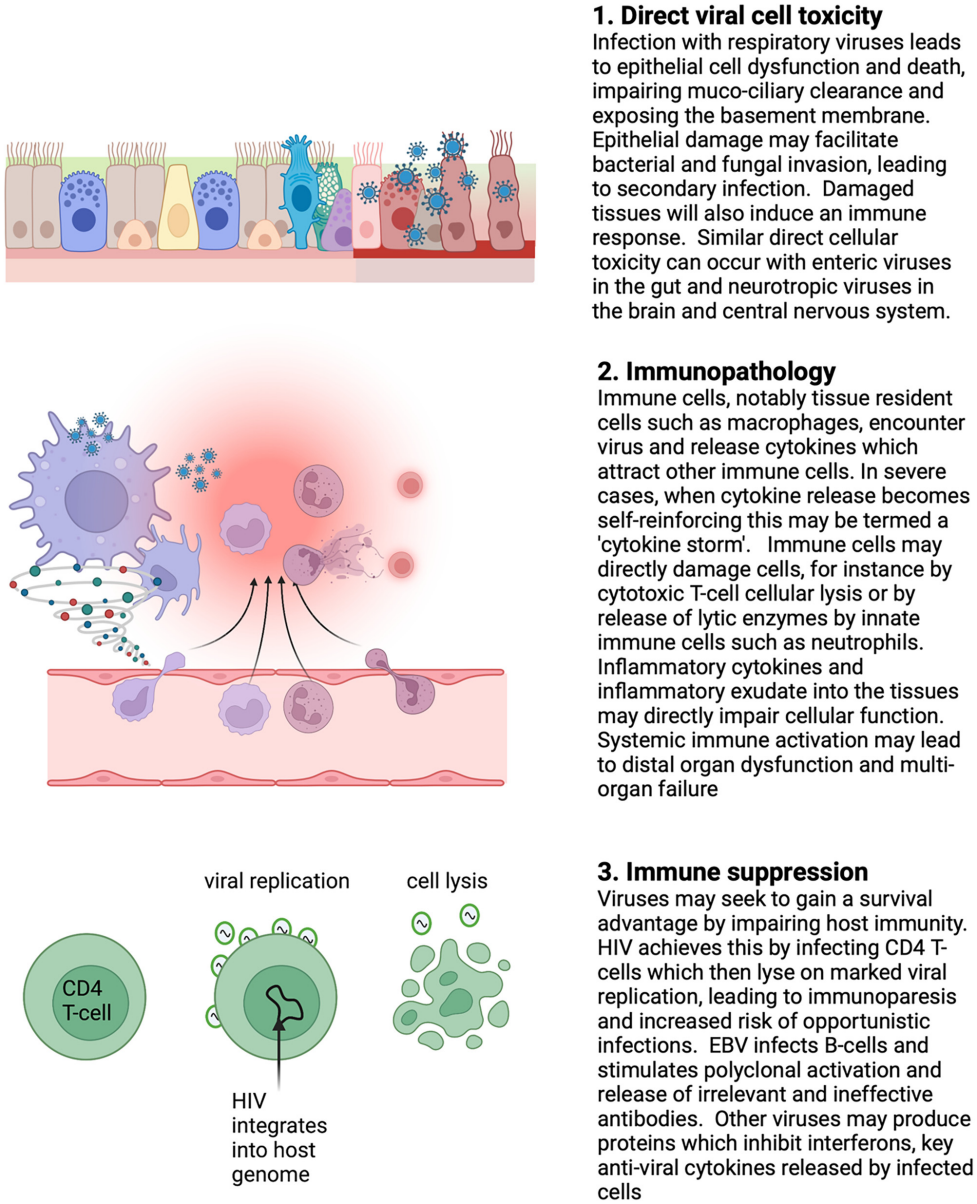
Examples of mechanisms of viral disease causation. Viruses may cause direct cellular damage and lysis (panels 1 and 3) or may induce host tissue impairment via immune activation and immunopathology (panel 2). In many cases the disease caused will arise from a combination of these effects, coupled with secondary infection or effects of secondary organ failure. These mechanisms are for illustration, an exhaustive list of viral pathogenesis is beyond the scope of this article. Figure made with BioRender.com.

Immune responses to viral infections are critical to the pathological features seen and Fig. 1 illustrates some of the mechanisms. Interactions between the virus itself, host genetic factors and comorbid conditions combine to influence the clinical presentation [1, 2].

## Respiratory viruses

Respiratory viruses come from a range of different viral families [3] that share tropism for cells within the respiratory tract. These viruses are commonly detected in patients with severe community‐acquired pneumonia, observed in 30–60% of cases [4, 5]. However, their role as causative pathogens remains incompletely understood [6]. Such viruses may present as co‐infecting pathogens with bacteria [4] or even as incidental detections in patients without clinical pneumonia [6]. The severity of illness arising from viral infection will vary from patient to patient, influenced by the virus itself; genetic predisposition to severe illness; age and comorbid conditions (e.g. immunosuppression, diabetes, chronic cardiac, respiratory and liver disease); pregnancy; and morbid obesity [7, 8]. While the most pathogenic viruses, such as influenza and SARS‐CoV‐2, are widely recognised in the intensive care unit (ICU), it is important to look for other viruses when evaluating patients with respiratory infections and failure, especially in children and patients with suppressed immunity [3]. There are a range of methods available to detect respiratory viruses, including direct antigen tests, nucleic acid amplification tests, viral culture and serology. Direct antigen tests, such as lateral flow assays for individual viruses have the advantages of speed and point‐of‐care use but are generally less sensitive than nucleic acid amplification assays. These are increasingly available as multiplexed and even point‐of‐care operable assays [9]. As respiratory viruses tend to be confined to the respiratory tract, and greatest yield is found at the site of pathology, with lower respiratory tract samples preferred for pneumonia diagnosis [10, 11]. Although commonly thought of as community‐acquired infections, viral illnesses can also be contracted in hospital [12]. Routine use of viral testing as part of broad‐spectrum diagnostic work‐up may help limit inappropriate or unnecessary antibacterial use [13].

### Influenza

Influenza viruses from alpha and beta (usually denoted A and B) genera make up the majority of human influenza infections, although gamma (denoted C) causes sporadic outbreaks of mostly mild illness [14]. Influenza A viruses infect other animals such as pigs and various bird species. Influenza A frequently mutates and recombines, leading to seasonal epidemics and sporadic pandemics arising from antigenic shift [15]. Influenza B is largely restricted to humans and is considered a more stable virus with fewer mutations [16]. However, influenza B does also undergo antigenic drift [15] with periodic epidemics, and constitutes approximately a quarter of global cases [17]. The clinical presentations of influenza A and B virus are indistinguishable [18]; both are associated with a high frequency of bacterial co‐infection at presentation, affecting around 35% of patients who require ICU treatment [19]. Although the evidence base is largely restricted to observational studies, the use of neuramidase inhibitors is currently recommended in critically ill patients with influenza [20], alongside testing for and empiric treatment of bacterial co‐infection. Although bacterial co‐infections have long been recognised in influenza, there is growing evidence of fungal co‐infection in the form of pulmonary aspergillosis [21] and a low threshold for investigation and treatment is required.

### Coronaviruses

Coronaviruses fall into four genera (alpha, beta, gamma and delta) with alpha and beta containing the human pathogenic viruses. Although seasonal coronaviruses, such as OC43, HKU1, 229E and NL63, can occasionally cause severe disease, this tends to occur in immunosuppressed patients [22]. The severe acute respiratory syndrome‐related viruses (sarbecovirus) SARS‐CoV, SARS‐CoV‐2 and merbecovirus MERS‐CoV have far greater pathogenic potential. However, as the SARS‐CoV‐2 pandemic has demonstrated, these viruses can produce a wide spectrum of illness from asymptomatic carriage to life‐threatening organ failure. The management of severe SARS‐CoV‐2 infection is now underpinned by one of the largest and most developed evidence bases found in critical illness, with established roles for immunomodulatory therapies (corticosteroids, interleukin 6 antagonists, Janus kinase inhibitors); recombinant antibodies seronegative patients; and non‐invasive respiratory support [23]. Antiviral therapies (such as remdesivir) can reduce disease progression in high‐risk patients early in their infection [23]. These drugs play little role, however, in the management of most patients who are admitted to ICU as hypoxaemia tends to occur late in the course of disease. While remdesivir may be of use in critically immunosuppressed patients, this is not based on firm evidence [24]. Although community‐acquired bacterial co‐infection is relatively rare among patients with severe COVID‐19, ICU‐acquired infections (bacterial [25, 26] and fungal [27]) are very common. Clinicians should always refer to evolving management guidance for COVID‐19 as new data continues to accumulate [23].

### Other respiratory viruses

There are a range of other respiratory viruses, including human metapneumovirus, para‐influenza, rhinovirus and respiratory syncytial virus. These are well established childhood pathogens, especially in infants, as a frequent cause of respiratory failure secondary to bronchiolitis [28]. Adenoviruses may provoke disease across a range of tissues, including the respiratory tract. Their highly contagious nature can lead to outbreaks and although usually mild, adenoviruses can produce severe disease even in immunocompetent adults [29]. While these non‐influenza respiratory viruses may be detected in up to a quarter of adult patients hospitalised with community‐acquired pneumonia [6, 30], the pathogenic attribution of pneumonia to these organisms alone is challenging. The syndromic presentation of severe disease in adults is often clinically indistinguishable from influenza or COVID‐19 and requires molecular testing for confirmation [31]. Immunodeficient patients and the elderly are at risk of severe diseases from these viruses, and hospital‐acquired transmission can occur [12, 31, 32]. The use of antivirals, most commonly the broad antiviral compound ribavirin, is not underpinned by strong evidence for these infections. Further, these drugs are not without side effects [33] and use should be guided by expert virological advice.

### Respiratory viral disease syndromes

Respiratory viral infections may present to ICU in several ways, often as an exacerbation of a pre‐existing condition such as chronic obstructive pulmonary disease and acute severe asthma. These diseases have well established treatment pathways, which include corticosteroids and (for chronic obstructive pulmonary disease) routine use of antibiotics [34, 35]. Where viruses are detected in the absence of co‐infecting bacteria, antibiotics may be avoided or rapidly de‐escalated. Given the high frequency of viral precipitants of exacerbations [36], and the clear evidence in favour of the use of corticosteroids in both exacerbated chronic obstructive pulmonary disease and asthma [34, 35], viral infections do not appear to be a contraindication in this setting. However, given the known adverse effects of steroids, their systemic use should be for as short a duration as possible [37].

Acute respiratory distress syndrome (ARDS) is a common presentation of severe respiratory infection necessitating ICU admission. Infectious pneumonia, of which viral infections make up approximately one‐third of cases, is the most common precipitant of ARDS [38]. Acute respiratory distress syndrome induced by viral infection should be managed using standardised guidelines including use of lung‐protective ventilation; early prone positioning in severe disease; and avoidance of fluid overload [39]. The use of corticosteroids in ARDS arising from viruses other than SARS‐CoV‐2 remains controversial, with conflicting results from trials in both ARDS [40, 41] and pneumonia [42, 43].

Non‐pulmonary complications of respiratory viruses have been widely described in the context of COVID‐19 and influenza. Organ failure may arise from the systemic inflammation triggered by viral infection, in a manner analogous to other sepsis syndromes [44]. However, direct viral pathology can also lead to cardiac failure through myocarditis and myocardial infarction, rhabdomyolysis and encephalitis. Viral infections can also trigger autoimmune responses. Both acute inflammatory syndromes such as Guillain–Barre syndrome (see post‐viral syndromes below) and chronic autoimmune diseases such as type‐1 diabetes have been linked to viral infections.

## Blood‐borne viruses

Blood‐borne viruses consist of a group of viruses spread via body fluids, most commonly blood and genital tract secretions, although other body fluids can transmit virus too. The most well‐known and prevalent of these are human immunodeficiency virus (HIV) and hepatitis B and C viruses (HBV and HCV respectively). Diagnosis of these infections is usually by serology in the first instance, with polymerase chain reaction assays for viral nucleic acids to look for active viral replication or as the primary test in immunocompromised patients who may not seroconvert.

### Human immunodeficiency virus

The advent of combination anti‐retroviral therapy (cART) has significantly altered the prognosis of HIV infection. The British HIV association guidelines undergo regular review and updates can be found at https://www.bhiva.org/guidelines. With the improvements in life‐expectancy associated for patients living with HIV, and related outcomes with non‐HIV infected patients with similar presentations, HIV status alone should not be used to decline ICU admission [45]. Anti‐retroviral drugs, however, have a wide range of potential side effects. Most notably for the intensivist is the association between nucleoside analogues and lactic acidosis mediated by mitochondrial toxicity [46], although this occurs much less frequently with modern nucleoside and nucleotide analogues. Anti‐retroviral drugs also have a wide range of drug–drug interactions, https://www.hiv‐druginteractions.org is a useful resource to check these. Where possible, existing cART should be continued in patients with HIV admitted to ICU, but specialist pharmacist advice on routes of administration and drug interactions may be required.

De‐novo presentation to ICU with HIV is rare in high‐income countries, although does occur, and is of greater prevalence in populations with limited access to healthcare including homeless individuals and undocumented migrants. When patients present with severe infections, especially when these involve opportunistic organisms, a HIV test is indicated and should be performed in the patient's best interests if they are unable to give explicit consent. Patients with a new diagnosis of HIV‐infection, especially if accompanied by low CD4‐T‐cell counts (<250.μl^−1^ blood) are at risk of opportunistic infections across multiple sites and should be carefully screened for such infections [45]. In such cases, early guidance should be sought from virology and/or infectious disease specialists.

The question of when to start cART in de‐novo HIV‐infection diagnosis in ICU remains uncertain and needs to be carefully managed with specialist teams [45, 47]. The use of cART can induce initial worsening of opportunistic infections, and can also induce systemic inflammation, lung injury collectively known as immune reconstitution syndrome [48]. The suggested approach is to ensure opportunistic infections are treated adequately before initiation of cART, with at least a 4‐week delay in cases of central nervous system tuberculosis or cryptococcal infection. Primary severe HIV infection, HIV encephalitis and multifocal leucoencephalopathy should prompt immediate cART initiation once opportunistic infections have been ruled out [45].

### Hepatitis viruses

Viral hepatitis may present to ICU as acute infection that requires urgent anti‐viral treatment. Treatment guidelines are available from the European Association for the Study of the Liver [49, 50]. Reactivation of hepatitis B can occur in patients receiving immunosuppressive medications, where again, anti‐viral treatment is indicated. However, the most common presentation is with decompensated chronic liver failure secondary to established cirrhosis from chronic infection. In this latter context, management should be as per guidelines for the management of decompensated chronic liver disease and acute‐on‐chronic liver failure, with expert gastroenterology or hepatology advice. Anti‐viral therapy is used in an attempt to promote viral clearance even when there is established cirrhosis, and patients may present to ICU due to complications of these treatments. Hepatitis C is treated with direct acting anti‐viral therapy, which is usually well tolerated [49]. Hepatitis B treatment is dependent on various factors including the stage of disease; viral load and degree of hepatocellular damage [50]. When pegylated interferon alpha 2 is used, complications may, rarely, lead to ICU admission.

## Enteric viruses

Multiple viruses demonstrate tropism for gastrointestinal cells, leading to infection via the orogastric route, producing gastrointestinal pathology. This group of viruses arise from different families including norovirus (a calicivirus); rotavirus; human astrovirus; hepatitis A (a picornavirus); hepatitis E (a hepevirus); and enteroviruses including poliovirus. Other viruses with a primary tropism for respiratory epithelium may also produce enteral infection with gastrointestinal symptoms, including coronavirade such as SARS‐CoV‐2 and enteroviruses. Infection with these viruses typically present with an acute gastroenteritis, with vomiting and watery diarrhoea. This may be difficult to distinguish from bacterial or toxic gastroenteritis clinically. Molecular testing of stools is the most reliable method to determine the causative pathogen [51]. Admission to ICU may be required if diarrhoea causes severe dehydration. Spread to extra‐intestinal tissues may cause specific syndromes which can also result in ICU admission. Classically, polio produces a flaccid paralysis which may require ventilatory support, indeed it was a polio epidemic that is attributed to the first developments of the ICU [52]. Other enteroviruses, most notably enterovirus D86, are also associated with acute flaccid paralysis [53]. Any case of suspected enterovirus‐associated acute flaccid paralysis should be discussed with the local health protection team urgently. The enteric hepatitis viruses can, as their names suggest, induce hepatitis although this is usually self‐limiting and is a rare cause of fulminant or acute liver failure necessitating ICU admission [54].

## Herpesviridae infections and reactivations

Herpesviridae is a family of double‐stranded DNA, enveloped viruses. The eight viruses known to infect humans are: herpes simplex virus (HSV) 1 and 2; herpes (varicella) zoster virus; cytomegalovirus (CMV); Epstein‐Barr virus (EBV); and human herpes virus 6, 7 and 8. Following primary infection, all these viruses enter a latent stage within the host cell and persist lifelong. It is estimated that at least 80% of adults are seropositive for HSV1, and approximately 50% seropositive for CMV [55]. Intensivists will encounter these viruses through conditions that present to ICU, but most commonly as reactivation in patients already in ICU. Latent infections can reactivate under conditions of physiological stress; immunosuppression; and acute critical illness [56]. Reactivation may be asymptomatic but can also produce serious illness such as encephalitis or malignant transformation [56].

### Herpes simplex virus 1

Although many patients infected with HSV1 will be asymptomatic, primary HSV1 can present as gingivostomatitis or pharyngitis, and painful vesicular lesions may be observed. Of more relevance to ICU clinicians is HSV encephalitis, which may occur as both a primary infection (most commonly in children and young people) or following reactivation of latent infection [57]. This is most reliably diagnosed by molecular testing of the cerebrospinal fluid.

Diagnosis may be supported by neuro‐imaging (MRI) with characteristic temporal horn involvement [57]. Treatment is with the antiviral acyclovir, started upon clinical suspicion rather than delayed pending diagnostic confirmation to limit cerebral damage [58].

Herpes simplex virus 1 is commonly detected in the respiratory secretions of ventilated patients, and is associated with severity of illness, duration of ventilation and severity of critical illness‐induced immunoparesis [59]. In most cases this is thought to reflect viral reactivation due to loss of host‐immune control of virus, and it is rare to find convincing evidence of herpes pneumonitis (cytological evidence of viral cell inclusions; positive HSV viral cell culture; and pneumonitis without other explanation). The use of antiviral treatment in these patients is not generally recommended and there is a lack of randomised trial evidence to support use [60]. However, in a retrospective study, patients with high viral load (>105 HSV copies.ml^−1^) detected by bronchoalveolar lavage sampling showed marked improvement with acyclovir compared with patients with low viral load [61], suggesting that there may be patients in whom antiviral therapy is beneficial. Herpes simplex virus 1 viraemia is also often used as a trigger for treatment. Immunocompromised patients, especially those with advanced HIV infection, transplant recipients and neutropaenic patients are at increased risk of HSV1 infection and prophylaxis may be used, although these patients are also at increased risk of developing acyclovir resistant virus. Herpes simplex virus 2, which is predominantly spread by genital contact, may produce similar presentations to those arising from HSV1 but is rare outside at‐risk populations (e.g. neonates and patients with immunocompromise).

### Cytomegalovirus

Similar to HSV, CMV often causes an asymptomatic primary infection in immunocompetent individuals but can cause disease on primary infection or reactivation following drug or infection‐induced immunosuppression and in older patients with immunosenescence. Cytomegalovirus colitis, retinitis, hepatitis and pneumonitis are all reported and, while most common in immunocompromised patients, can occur in the apparently immunocompetent who are immunonaïve or immunosenescent. In mechanically ventilated, critically ill patients, CMV reactivation in respiratory secretions is common [62]. It is not clear, however, that the associations with prolonged length of ICU stay and mortality are causal and randomised trials of CMV treatment have not demonstrated benefit [63]. As with HSV, this lack of benefit may reflect a failure to identify patients with genuine CMV pneumonitis rather than incidental reactivation. Where patients are at high risk of CMV reactivation, such as solid organ transplantation from a CMV‐positive donor to a CMV‐negative recipient, antiviral prophylaxis with ganciclovir is indicated [64]. The presence of CMV viraemia or convincing evidence of CMV tissue pathology may require biopsy to distinguish from incidental reactivation and should prompt treatment. Ophthalmology review for CMV retinitis should be undertaken in patients with evidence of disseminated CMV infection, including CMV viraemia.

### Varicella zoster virus

Varicella zoster virus causes chicken pox as primary infection, and reactivation leads to shingles in the immunosuppressed and immunosenescent. While neither of these are likely to result in ICU admission, shingles can develop as a painful and distressing consequence of critical illness and may benefit from early antiviral treatment. Shingles can transmit infection to the immunonaïve or immunocompromised and careful attention to infection control is required. In immunocompetent but varicella zoster virus‐naïve adults, varicella zoster virus can cause a severe pneumonitis with consequent respiratory failure [65]. Acyclovir is used and prompt treatment may reduce severity of symptoms. The use of steroids remains uncertain and not backed by strong evidence [65]. Varicella zoster virus can also cause encephalitis and should be looked for in patients without other identified pathogens.

### Epstein‐Barr virus

Epstein‐Barr virus, in common with most herpesviridae, can cause mild, self‐limiting illness or asymptomatic infection. The most common symptomatic presentation is with infectious mononucleosis, characterised by fever, lassitude, lymphadenopathy and pharyngitis, with a concurrent peripheral blood lymphocytosis. Cytomegalovirus may also cause an illness to be hard to distinguish from EBV infection clinically. Primary EBV infection can be associated with splenomegaly, abnormal liver function tests and occasional splenic rupture. Epstein‐Barr virus, in common with a number of other viruses, can trigger haemophagocytic lymphohistiocytosis (HLH, see below) [66]. Epstein‐Barr virus is associated with a range of malignancies, notably Burkitt's lymphoma and post‐transplant lymphoproliferative disorder [64].

## Unusual and emerging viruses

The nature of intensive care is that patients frequently present with undifferentiated acute and critical illness. As such, unusual infections may present to intensivists sporadically, and maintaining an appropriate threshold of suspicion may reduce risks of delayed and missed diagnoses.

### Viral haemorrhagic fevers

Viral haemorrhagic fevers are a range of illnesses caused by a diverse group of viruses with varying, and sometimes overlapping, geographic distributions. With the increasing degree of global interconnectedness, viral haemorrhagic fevers may present to non‐endemic areas as imported fever or even in‐country transmission. Within the UK, viral haemorrhagic fevers are rare, with 113 cases notified to public health authorities between 1982 and 2020, averaging three cases a year [67]. However, the West African Ebola epidemic of 2013–2016 killed at least 11,000 people [68], and the annual burden of Lassa fever across the African continent is estimated to be between 100,000 and 300,000 cases [69], presenting a considerable global burden of disease.

Viral haemorrhagic fevers present with fever, malaise and vascular leak with hypovolaemia. Despite the name, the degree of haemorrhage is variable, and its presence is not a sensitive predictor of disease. Thrombocytopaenia, coagulopathy and leukopaenia are all features of viral haemorrhagic fevers, especially when disease is severe [70]. These features are all non‐specific and can also be seen in sepsis arising from other pathogens, including parasitic infections such as *Plasmodium falciparum*. It is therefore important to ensure a comprehensive travel history is taken from all patients presenting with acute febrile illness, including travel history of recent close contacts, and maintain an appropriate degree of suspicion in patients who are at risk. Within the UK, the Imported Fever Service is available to advise on appropriate testing after initial consultation with local microbiology, virology or infectious disease specialists. Guidance on management of suspected cases is available from the UK Health Security Agency [71]. Where viral haemorrhagic fever is suspected, it is important to maintain appropriate isolation and rigorous personal protective equipment standards to prevent further spread [71]. Patients with confirmed disease should be transferred to a high‐consequence infectious disease unit, with an extensive infection control exercise required with patient and sample contact tracing.

Management of viral haemorrhagic fevers is supportive, with fluid resuscitation, electrolyte replacement and ventilatory and non‐respiratory organ support as required. Ebola is the best studied viral haemorrhagic fever, following the 2013–2016 West African epidemic and subsequent 2018 Democratic Republic of Congo outbreak. Although mortality was high in these settings, the provision of advanced critical care was associated with an 82% survival rate [72]. Ebola is the only viral haemorrhagic fever with a specific therapy in the form of monoclonal antibodies which were proven efficacious in the trial by Mulangu et al. [73].

### Emerging viruses

As the recent outbreak of monkeypox (renamed in late 2022 as mpox by the World Health Organization) demonstrates, imported and emerging viruses are likely to be an increasing feature of clinical practice in a globalised world. Many viruses demonstrate the ability to cross species boundaries, and a substantial proportion of the global virome (global load of viruses) circulates within non‐humans. Close interactions between humans and animals can lead to the emergence of novel viruses, and recirculation of rarer viruses. Many viruses can be carried by invertebrates, and a history of tick bites or mosquito bites followed by a non‐specific febrile illness and encephalitis may be indicative of an arboviral infection. People who are in close contact with mammals, including livestock farmers, animal breeders and animal technicians, are at risk of acquiring viruses, for instance the lethal European outbreak of squirrel bornavirus encephalitis occurred among three breeders of exotic squirrels [74]. Other animal‐borne viruses which may infect humans include rabies, acquired from infected bats or canines in endemic countries, and hantaviruses which are contracted from infected rodents. Other emerging, animal‐associated viruses which have caused outbreaks in recent years include the Nipha and Hendra viruses [75] that, similar to SARS‐CoV‐2, likely have a reservoir in bats. These less common viruses emphasise the need for careful exposure history taking and continued search for causative agents when the initial investigations are inconclusive. Previous clinical experience with these viruses is likely to be limited, even among experts. Dealing with the uncertainties of diagnosis, risk to staff and optimal management make such situations challenging to manage.

## Post‐viral syndromes

Viral infections can induce pathology not only in their direct pathology and immediate host response but can also induce indirect organ damage by triggering later immune responses. Haemophagocytic lymphohistiocytosis was originally described as a genetically mediated primary autoimmune disease found in children. However, it is now well recognised that secondary HLH can be triggered by a range of stimuli, including viral infections. Whilst EBV is the most common reported viral trigger, accounting for 43% of cases, HLH has been associated with a range of herpesviridae as well as adenovirus, HIV, influenza [76] and SARS‐CoV‐2 [77]. Haemophagocytic lymphohistiocytosis presents with systemic inflammation, organ failure and elevated cytokines and can be difficult to distinguish from sepsis without additional investigations. The elevated ferritin, multiline cytopaenias and abnormal liver function tests included in diagnostic criteria are non‐specific, and definitive diagnosis may require detection of elevated soluble IL‐2 receptor (sCD25) and bone marrow aspirate confirming haemophagocytosis (ingestion of erythrocytes, platelets or leukocytes) by macrophages. Management of secondary viral‐induced HLH is not based on firm evidence and expert haematological and virological advice should be sought when this condition is suspected.

Viral infections may also trigger auto‐immune diseases. A prominent example which leads to frequent admission to ICU is Guillain–Barré syndrome, where up to one‐third of affected patients will require mechanical ventilation [78]. The flaccid paralysis that characterises Guillain–Barré syndrome arises from immune cells which target peripheral neurons or their supporting cells as a result of recognising ‘self’ antigens. In around two‐thirds of cases this is triggered by an acute respiratory or enteric infection, a substantial proportion of which will be viral in origin [78].

## Infection control

Nosocomial spread through exposure to body fluid containing viable virus is an important clinical risk to address. The methods of prevention of transmission are dependent on the route of spread and are covered in detail in infection control guidance [79]. Respiratory viruses spread predominantly by droplet and aerosol, and therefore protection consists of respiratory personal protective equipment including FFP3 or an equivalent respirator to reduce near‐field spread when aerosols are generated, and isolation rooms with effective ventilation or air filtration [80] to reduce far‐field spread. Enteric viruses can also spread by droplet if vomiting is present and direct faecal‐oral contact. Protection is by isolation room and use of contact precautions with a fluid‐resistant surgical mask if vomiting is present. Members of staff infected with these respiratory and enteric viruses should be excluded from work until symptoms have receded to limit risk to patients.

Blood‐borne viruses are spread by contact between the infected body fluid and another person's mucosa or broken skin. Prevention of infection here is by contact precautions and safe handling of sharps. If blood is likely to be aerosolised (e.g. during a surgical procedure) then fluid‐resistant surgical mask and eye protection should be worn. All infection control is underpinned by meticulous hand hygiene, changing of personal protective equipment between patients and excellent environmental cleaning with a particular emphasis on hand‐touch sites. Infection control extends beyond immediate patient care and includes contact tracing during outbreak situations or with notifiable diseases to seek to prevent onward transmission and to ensure contacts are appropriately monitored and managed.

## Areas for future research

There are a range of unanswered questions regarding viral infections in the ICU. The most pressing relate to the extent to which therapeutics with proven benefit in COVID‐19 treatment can be applied to ARDS arising from other respiratory viral infections. The role of molecular diagnostics in detecting or excluding bacterial co‐infection and the impact on antimicrobial prescribing also requires urgent exploration. The third area of research interest is to define which patients with evidence of respiratory herpesviridae reactivation will benefit from antiviral therapy.

## Conclusions

Viral infections form a significant proportion of the ICU workload, and commonly present with non‐specific syndromes that require viral testing for formal diagnosis. Diagnostic testing should be informed by the presenting syndrome and careful history taking, with due attention to recent sick contacts, animal contacts and travel history. Secondary complications of viral infections, including co‐infection and ICU‐acquired infection need to be distinguished from primary viral pathology as these will require specific therapy with appropriate antimicrobials. The range of antiviral therapies is limited, and their optimal use is well defined only in a narrow number of infections caused by the most prevalent viruses. Consultation with microbiology, virology or infectious diseases specialists can help when unusual or complicated infections are suspected and vital when viral haemorrhagic fevers are suspected. With the challenges of climate change, habitat invasion by humans and global interconnectedness, viral illness from both well established and emerging pathogens are likely to challenge intensivists for the foreseeable future.
